# Still and Silent: When Psychosis Looks Like Conversion

**DOI:** 10.1192/j.eurpsy.2026.12152

**Published:** 2026-07-31

**Authors:** M. A. Andreo-Vidal, M. Calvo-Valcárcel, C. Rodríguez-Valbuena, J. C. Fiorini-Talavera, A. San Román-Uria, B. Rodríguez-Rodríguez, G. M. Medina-Ojeda

**Affiliations:** Psychiatry, Hospital Clínico Universitario de Valladolid, Valladolid, Spain

## Abstract

**Introduction:**

The differential diagnosis between conversion disorders and psychotic episodes can present a significant clinical challenge. Both conditions may share striking symptoms such as mutism, immobility, altered states of consciousness, and bizarre behavior, which can lead to diagnostic confusion—especially in emergency settings.

**Objectives:**

This case study aims to analyze the atypical clinical presentation of conversive symptoms in a patient in the emergency room.

**Methods:**

A review of the literature on conversive symptomatology which may occur in psychosis.

**Results:**

A 50-year-old woman with a psychiatric history since age 16, including multiple episodes with psychotic and conversive features—four requiring hospitalization. Her relatives report previous diagnoses of schizophrenia and bipolar disorder, but no records are available due to her international relocation. She is taking quetiapine 50 mg and oxcarbazepine 300 mg.

She arrives at the emergency room with her husband due to mutism. Organic causes are ruled out. On examination: mutism, perplexed gaze, failure to follow commands, and oppositional rigidity that varies and decreases with distraction. She imitates guided movements without difficulty; Hoover’s sign is positive. A conversion disorder is suspected. Diazepam 15 mg and lorazepam 2 mg are administered.

Her husband reports increased anxiety, insomnia, mutism, and immobility over the past week, alternating with periods of normal behavior. Two weeks prior, she became distressed about returning to work. He states that similar episodes have occurred under stress and resolved within days with benzodiazepines.

After 24 hours, she is able to speak and move, but appears confused, with no memory of the episode. She describes a sense of strangeness, makes interpretive remarks, and shows loose associations. Speech is vague and poorly structured. She is admitted and started on olanzapine 10 mg and amisulpride 800 mg.

**Conclusions:**

This clinical case illustrates an acute presentation initially characterized by mutism and immobility, compatible with a possible conversion disorder. However, the partial response to benzodiazepines and the subsequent emergence of psychotic symptoms suggest a different or more complex etiology.

One possibility is a psychotic episode with pseudoconversion symptoms—behaviors that mimic a conversion disorder but are actually expressions of disrupted thought processes. Another potential diagnosis is a brief psychotic disorder with catatonic features. Additionally, a polymorphic acute psychotic disorder (as defined in ICD-10) could explain the abrupt onset, symptom fluctuations throughout the day, and the presence of atypical features.

This is a diagnostically challenging case in the emergency setting, due to its atypical presentation and lack of information regarding the patient’s psychiatric history. Access to her medical records and longitudinal observation would be essential for reaching a clearer diagnostic conclusion.

**Disclosure of Interest:**

None Declared

